# Mesh-free prepectoral breast reconstruction: a safe and effective alternative

**DOI:** 10.3389/fonc.2026.1816651

**Published:** 2026-05-20

**Authors:** Jun Zhang, Hai-Kuo Yu, Zi-Fan Liu, Ran An, Zhi-Hao Yu, Li Zhang

**Affiliations:** 1 Thyroid and Breast Medical Center, Weifang People’s Hospital, Shandong Second Medical University, Weifang, Shandong, China; 2 Department of Anesthesiology, Weifang People’s Hospital, Shandong Second Medical University, Weifang, Shandong, China; 3 Department of Breast Disease, Weifang Maternal and Child Health Hospital, Weifang, Shandong, China; 4 The First Department of Breast Cancer, Tianjin Medical University Cancer Institute and Hospital, National Clinical Research Center for Cancer, Tianjin, China

**Keywords:** capsular contracture, complications, cost-effectiveness, mesh-free, prepectoral breast reconstruction, quality of life

## Abstract

**Objective:**

Prepectoral breast reconstruction (PBR) offers several advantages, including reduced postoperative pain, fewer animation deformities, shorter operative time, better aesthetic outcomes, and greater patient comfort, and has gradually become an important option for post-mastectomy reconstruction. However, most current prepectoral reconstruction procedures still rely on mesh (such as acellular dermal matrix [ADM] or synthetic mesh) to enhance soft tissue coverage and reduce the risk of implant-related complications, such as capsular contracture and implant exposure. The use of mesh, however, also introduces additional concerns, including high cost, risk of infection, and foreign body reactions. Therefore, this study aims to compare two surgical approaches—prepectoral breast reconstruction with mesh (PBR with mesh) and prepectoral breast reconstruction without mesh (PBR without mesh)—in breast cancer patients, evaluating their clinical outcomes and aesthetic results, and to summarize the advantages of mesh-free prepectoral breast reconstruction.

**Methods:**

This study retrospectively analyzed the clinical data of 246 patients who underwent PBR between June 2023 and December 2024. Patients were divided into two groups according to the surgical approach: the mesh-free group (128 cases, including 26 who received postoperative radiotherapy) and the mesh group (118 cases, including 25 who received postoperative radiotherapy). Baseline characteristics, perioperative indicators (such as operative time and blood loss), and postoperative complications (including infection, capsular contracture, and implant loss) were thoroughly evaluated and subjected to cost analysis. A subgroup analysis was performed specifically for the 51 patients who received postoperative radiotherapy (26 in the mesh-free group and 25 in the mesh group), with particular focus on comparing the incidence of capsular contracture between the two groups. In addition, the BREAST-Q questionnaire was used to systematically assess patients’ postoperative quality of life and satisfaction.

**Results:**

Regarding major complications, no statistically significant differences were observed between the mesh-free and mesh groups: infection rate (3.13% vs. 5.08%, p=0.527), severe capsular contracture rate (2.34% vs. 4.24%, p=0.486), wound dehiscence (2.34% vs. 3.39%, p=0.713), and implant loss rate (2.34% vs. 3.39%, p=0.713) (all p>0.05). Regarding perioperative indicators, there was no statistically significant difference in intraoperative blood loss and postoperative drainage volume between the two groups. The operation time in the non-patch group was significantly shorter than that in the patch group (107.50 [95.00, 120.00] vs. 125.00 [115.00, 135.00] minutes, p<0.001) and lower hospitalization costs (42,242.36 [39,999.14, 44,628.14] RMB vs. 60,083.01 [57,592.06, 63,443.36] RMB, p<0.001). In the subgroup analysis of 51 patients who received postoperative radiotherapy, the incidence of severe capsular contracture was higher in the mesh group compared with the mesh-free group (20% vs. 7.7%), although the difference did not reach statistical significance (p=0.193). BREAST-Q results showed no significant differences between the two groups in Satisfaction with breasts (54.00 [53.00, 56.00] vs. 53.50 [52.00, 55.00], p=0.051), Psychological well-being (39.50 [38.00, 41.00] vs. 39.00 [37.00, 40.00], p=0.092), or Sexual well-being (19.00 [18.00, 20.00] vs. 19.00 [18.00, 19.25], p=0.179). However, the mesh-free group had significantly higher scores in Physical well-being: chest (31 [29, 32] vs. 27 [25, 33.25], p=0.005).

**Conclusion:**

In selected patient populations, mesh-free prepectoral breast reconstruction is a safe and cost-effective option.

## 1 Introduction

The global annual incidence of breast cancer exceeds 2 million cases, ranking first among malignant tumors in women and representing the leading cause of cancer-related death, thereby posing a serious threat to women’s physical and mental health (1). Traditional radical mastectomy often results in substantial physical trauma and psychological stress for patients. Consequently, surgeons have been striving to balance effective disease control with patients’ pursuit of higher quality of life. Prosthetic breast reconstruction, compared with traditional surgical approaches, offers advantages such as less trauma, preservation of breast contour, reduced psychological burden, and greater potential for psychological and social recovery. Furthermore, unlike autologous tissue reconstruction, it avoids donor-site morbidity, preserves physical function, allows faster postoperative recovery, and is associated with fewer complications. Thus, it has been increasingly performed in recent years (2–4). The evolution of implant pocket placement has progressed from prepectoral (subcutaneous) implants to subpectoral implants with expanders, and more recently to mesh-assisted prepectoral reconstruction. Multiple studies have confirmed the safety of the prepectoral approach, though most rely on mesh support (4–11). Given the high cost and increased infection risk associated with mesh, interest has grown in exploring mesh-free prepectoral reconstruction (12–16). This retrospective study included 246 patients and aimed to evaluate the feasibility, safety, and clinical outcomes of mesh-free prepectoral breast reconstruction in selected patient populations, in order to provide evidence for optimizing surgical strategies.

## 2 Materials and methods

### 2.1 Patient selection

This study retrospectively analyzed 246 patients who visited the Department of Breast Tumors I at Tianjin Cancer Hospital and underwent prepectoral implant-based breast reconstruction between June 2023 and December 2024 (**Table 1**). Inclusion criteria: patients who underwent subcutaneous mastectomy combined with immediate prepectoral implant-based breast reconstruction. Exclusion criteria: incomplete follow-up data or refusal to participate in follow-up; implantation of a soft tissue expander instead of a prosthetic implant. The study protocol was approved by the Ethics Committee of Tianjin Cancer Hospital (Approval No.: bc20240330), and all patients met the following requirements: ①Early-stage breast cancer patients who were not suitable for, or did not wish to undergo, breast-conserving surgery; tumor size <5 cm (clinically or radiologically); clinically negative axillary lymph nodes or clinically positive but biopsy-negative nodes. ②Adequate skin condition with sufficient subcutaneous fat thickness (In general, MRI measurements show that the parasternal subcutaneous fat is ≥1 cm, and the fat layer above the mass is ≥0.5 cm). ③On MRI evaluation, the tumor shows no invasion of the skin, subcutaneous tissue, or chest wall, and there are no prominent long spiculations extending toward the skin or subcutaneous fat.

**Table 1:** Clinical data of the study patients.

Variable	PBR without mesh (N = 128)	PBR with mesh (N = 118)	P Variable
Age(years)			
Mean Range	44.08 ± 7.67	44.01 ± 9.22	0.949
Neoadjubant chemotherapy,n(%)	15.6	20.3	0.335
Tumor location,n(%)			
Upper Outer Quadrant	50.8	50.8	0.275
Upper Inner Quadrant	17.2	12.7	
Lower Outer Quadrant	18.8	13.6	
Lower Inner Quadrant	4.7	9.3	
ALND, n (%)	18.0	21.2	0.525
pT staging, n (%)			
pT1	39.1	51.7	0.135
pT2	56.3	44.1	
pT3	4.7	4.2	
Radiation after surgery, n (%)	20.3	21.2	0.866
Degree of tumor differentiation, n (%)			
G0	17.2	22.0	0.079
G1	10.9	19.5	
G2	49.2	34.7	
G3	22.7	23.7	
Pathological type, n (%)			
ER(+), HER-2(+)	17.19	9.32	0.225
ER(+), HER-2(-)	68.75	72.19	
ER(-), HER-2(+)	6.25	6.78	
ER(-), HER-2(-)	7.81	12.71	
Ki67	20(15,40)	30(18.75,41.25)	0.122

ER, estrogen receptor; PR, progesterone receptor; HER2, human epidermal growth factor receptor type 2; SLNB, sentinel lymph node biopsy; ALND, axillary lymph node; dissection; pT staging, pathological tumor staging; pN staging, pathological lymph node staging;PBR, Prepectoral Breast Reconstruction;.

Patients were divided into a mesh group and a mesh-free group according to whether a mesh (TiLop Bra breast soft tissue reinforcement mesh; pfm medical group, Germany) was used intraoperatively. All patients provided written informed consent preoperatively, and postoperative satisfaction was assessed using the BREAST-Q questionnaire. Follow-up was conducted through outpatient visits and telephone contact until December 2025.

### 2.2 Surgical procedures

#### 2.2.1 Preoperative marking

The patient was positioned standing, and the breast contour, inframammary fold, tumor surface projection, and estimated sentinel lymph node location were marked.

#### 2.2.2 Surgical positioning and preparation

The patient was placed in a supine position with the ipsilateral arm abducted to 90° and the shoulder elevated. The surgical field was disinfected to include the affected limb, allowing the arm to be raised to forehead level during surgery to facilitate Level III lymph node dissection and provide adequate exposure of the axilla during endoscopic procedures.

#### 2.2.3 Implant selection

The choice of implant (Mentor MemoryGel Xtra Breast Implants; Mentor Worldwide LLC, USA) was determined based on preoperative measurements (linear measurement method) and the size of the excised glandular tissue intraoperatively (weighing method). Most implants were textured anatomical, with a few being textured round. No further analysis was performed due to the extremely low usage of textured round implants.

Linear measurement method: ①Breast base diameter: Using calipers, measure the distance from 1.5 cm lateral to the midline to the anterior axillary line (X). The V-shaped skinfold caliper is used to measure lateral tissue thickness (Y) and medial tissue thickness (Z). Implant base diameter = X – (Y/2 + Z/2). ②Implant height (low, medium, full, extra-full): The distance from the midpoint of the sternal notch to the nipple is SN, and the distance between the nipples is NN. If SN – NN = 0–2 cm, a medium-height implant is selected; if SN – NN < 0, indicating lateral displacement of the breast and nipple, a low-height implant is chosen; if SN – NN > 2 cm, indicating medial and inferior displacement of the breast, a full- or extra-full-height implant is selected. ③Implant projection: For significant ptosis, loose skin, or desire for larger implant volume, a full projection implant is chosen; otherwise, medium or low projection implants are used.

Weighing method: After excision of the glandular tissue, the tissue is placed on a high-precision electronic scale to determine its weight, which approximates the required implant volume.

#### 2.2.4 Sentinel lymph node biopsy

A curved incision approximately 3 cm in length was made along the lateral edge of the breast, one finger breadth from the axillary skin crease (extended at both ends if axillary lymph node dissection was planned). Fifteen minutes before surgery, blue dye was injected subcutaneously along the edge of the areola. Sentinel lymph nodes were retrieved through the axillary incision, and the decision to perform axillary lymph node dissection was made based on intraoperative frozen section pathology results.

#### 2.2.5 Open prepectoral breast reconstruction procedure

The vast majority of patients underwent a radial incision on the lateral side of the affected breast. After skin incision, a subcutaneous injection of epinephrine saline (250 mL of 0.9% sodium chloride solution + 1 mL of 0.1% epinephrine) was administered. Skin flaps were dissected using scalpel and scissors within the preoperatively marked breast boundaries. Care was taken to preserve the periareolar ligament, especially medially—avoid excessive dissection toward the sternum for two reasons: first, there are perforating vessels in the medial second intercostal space; second, preserving the parasternal ligaments helps maintain the inframammary fold and cleavage structure. Inferiorly, the triangular fascial bundles and horizontal ligaments at the lower pole were protected, and the inframammary fold was preserved. During dissection of the retromammary space, the pectoralis major fascia was preserved to allow better breast mobility, and glandular tissue was excised completely from the superolateral side.

Before 2024, most prepectoral implant reconstructions in the department involved mesh reinforcement, typically using two techniques: ①Total coverage (wonton method): Two mesh sheets were selected, edges approximated and sutured to form a single large mesh that fully enveloped the implant. The four corners of the mesh were overlapped, and the first suture on the posterior side of the implant was performed using 3–0 absorbable sutures. The newly formed four corners were then sutured to the posterior mesh, and the edges were trimmed and closed to achieve complete coverage. ②Partial coverage (anterior wall coverage) method: This method had two variations. One involved covering only the anterior surface of the implant, while the posterior surface partially contacted the pectoralis major fascia; the mesh edges on the posterior surface were approximated using 3–0 absorbable sutures. The other involved extending the mesh, fixing the lower edge to the pectoralis fascia above the inframammary fold so that part of the posterior implant surface was covered. This method partially enveloped the anterior and posterior implant surfaces, ensuring that the implant surface directly under the incision was covered by mesh (This method was predominantly adopted by the surgeons). Since the second half of 2023, The surgeon currently rarely uses mesh, and the decision to use mesh was made intraoperatively after comprehensive clinical assessment, with the main indications for mesh placement being: ①Implant volume exceeding 355 cc ②Severe breast ptosis ③High skin tension at the incision site despite a relatively small implant volume.

One drainage tube was placed above and one below the implant, with the inferior tube exiting near the anterior axillary line at the inframammary fold, connected to high-negative-pressure suction. The incision was closed with intradermal sutures.

#### 2.2.6 Postoperative management

The implant was wrapped with sterile dressings to limit its movement and displacement, and an outer compression bra was applied with appropriate pressure until the drains were removed. The nipple was covered with hollow gauze to prevent pressure-induced ischemia. Prophylactic antibiotics were administered 0.5–1 hour before surgery and within 24 hours postoperatively to prevent infection. Drains were removed when the output remained below 15 mL/day for two consecutive days. After drain removal, patients could wear a regular bra without the need for a shaping or compression bra.

Postoperative adjuvant treatment, including chemotherapy, radiotherapy, targeted therapy, and endocrine therapy, was planned according to the patient’s final pathological results.

### 2.3 Follow-up and data analysis

#### 2.3.1 Data on patient age, tumor size on imaging, and routine postoperative pathological results were collected for both groups. Surgical outcomes, including operative time, intraoperative blood loss, total drainage volume in the first three days after surgery, total hospitalization costs, and complications such as infection, chest pain, wound dehiscence, and implant loss, were observed and compared between the two groups.

#### 2.3.2 The BREAST-Q questionnaire for breast cancer patients was used to assess breast satisfaction, psychosocial well-being, chest wall physical well-being, and sexual well-being.

#### 2.3.3 Follow-up data on survival and complications were collected using a combination of medical records, outpatient visits, telephone calls, WeChat, and questionnaires. Follow-up continued until December 2025.

### 2.4 Statistical methods

Continuous variables were described as mean ± standard deviation for normally distributed data or as median (first quartile, third quartile) for non-normally distributed data. Differences in patient age, operative time, hospitalization costs, BREAST-Q scores, blood loss, and routine pathological Ki-67 index were compared using the independent-samples t-test (for normally distributed data) or the Mann-Whitney U test (for non-normally distributed data). Categorical variables, such as lesion location, tumor stage, hormone receptor status, tumor histological grade, HER-2 status, receipt of neoadjuvant chemotherapy, and performance of axillary dissection, were presented as absolute numbers and percentages. Differences in baseline characteristics between the two groups were assessed using the chi-square test. All statistical analyses were performed using SPSS version 31.0.0.0(117) (IBM Corp., Armonk, NY, USA), and a p-value < 0.05 was considered statistically significant.

## 3 Results

### 3.1 Patient demographic characteristics

A total of 128 patients were included in the non-mesh group and 118 in the mesh group. There were no statistically significant differences between the two groups in terms of age, tumor stage, conventional pathological histological grade, tumor immunohistochemical status (ER, PR, Her-2, Ki-67 index), tumor location, and proportion of patients receiving neoadjuvant therapy. The median total hospitalization cost was significantly lower in the non-mesh group compared with the mesh group (42,242.36 [39,999.14–44,628.14] CNY vs. 60,083.01 [57,592.06–63,443.36] CNY, p<0.001). Postoperative radiotherapy was administered in 26 patients in the non-mesh group and 25 patients in the mesh group, with no significant difference between groups (**Tables 1**, **2**).

**Table 2:** Perioperative features of patients.

Variable	PBR without mesh (N = 128)	PBR with mesh (N = 118)	P Variable
Operation time (min)			
Median Q1–Q3	107.50(95.00,120.00)	125.00(115.00,135.00)	<0.001
Intraoperative blood loss (mL) Median Q1–Q3	50.00(50.00,70.00)	60.00(45.00,75.00)	0.081
Total drainage volume(mL) Mean Range	202.99 ± 60.55	198.44 ± 54.48	0.620
Hospitalization cost (yuan) Median Q1–Q3	42242.36 (39999.14,44628.54)	60083.01 (57592.06,63443.26)	<0.001

PBR Prepectoral Breast Reconstruction;.

### 3.2 Comparison of perioperative characteristics

There was no statistically significant difference between the two groups in the number of patients undergoing ALND (23 cases in the non-patch group and 25 cases in the patch group), intraoperative blood loss, and total drainage volume in the first three days postoperatively. The operative time was shorter in the non-mesh group [107.50 (95.00, 120.00) min] than in the mesh group [125.00 (115.00, 135.00) min], P < 0.001 (**Table 1**; **Table 2**).

### 3.3 Postoperative complications and oncological outcomes

There were no statistically significant differences between the two groups in terms of wound dehiscence, prosthesis loss (3 cases in the non-mesh group vs. 4 cases in the mesh group), surgical site infection (4 cases in the non-mesh group vs. 6 cases in the mesh group), and severe capsular contracture (3 cases in the non-mesh group vs. 5 cases in the mesh group) (**Table 3**). A subgroup analysis was performed among 51 patients who received postoperative radiotherapy. Although the rate of capsular contracture was higher in the mesh group than in the non-mesh group (20% vs. 7.7%, P = 0.193), the difference was not statistically significant (**Table 3**; **Table 4**).

**Table 3:** Postoperative complicatins.

Variable	PBR without mesh (N = 128)	PBR with mesh (N = 118)	P Variable
Wound infection, n (%)	3.13	5.08	0.527
Incision dehiscence, n (%)	2.34	3.39	0.713
Implant loss, n (%)	2.34	3.39	0.713
Severe capsular contracture, n (%)	2.34	4.24	0.486

PBR, Prepectoral Breast Reconstruction; Severe Capsular Contracture was defined as Baker grade III or IV.

**Table 4:** Comparison of severe capsular contracture in radiotherapy-treated patients: with vs. without mesh.

Variable	PBR without mesh (N = 26)	PBR with mesh (N = 25)	P Variable
Severe capsular contracture, n (%)			
Yes	2(7.7%)	5(20%)	0.193
No	24(92.3%)	20(80%)	

PBR Prepectoral Breast Reconstruction; Severe Capsular Contracture was defined as Baker grade III or IV.

As of December 2025, only patients in the mesh group experienced tumor recurrence, with one case of local recurrence and one case of axillary lymph node recurrence; therefore, further analysis was not performed.

### 3.4 Postoperative outcomes and BREAST-Q questionnaire results

**Figure 1:**
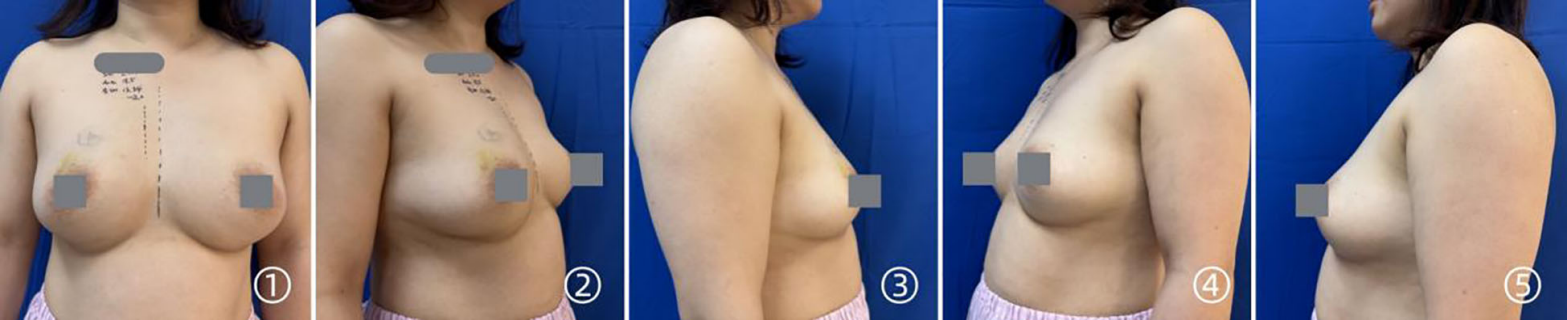
Preoperative breast appearance in a patient undergoing prepectoral prosthetic breast reconstruction without mesh.

**Figure 2:**
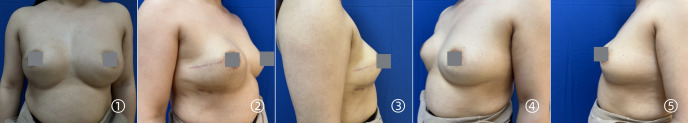
Breast appearance at 1 month after prepectoral prosthetic breast reconstruction without mesh.

**Figure 3:**
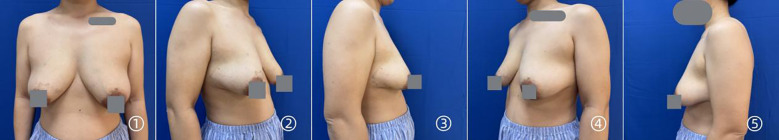
Preoperative breast appearance in prepectoral prosthetic breast reconstruction with mesh.

**Figure 4:**
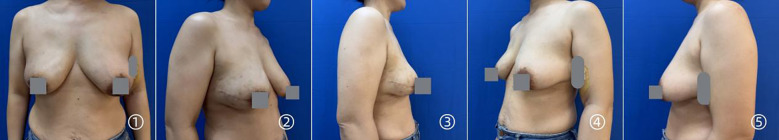
Breast appearance at 1 month after prepectoral prosthetic breast reconstruction with mesh.

Breast appearance preoperatively and postoperatively is shown in **Figures 1**–**4**. After 6 months postoperatively, all patients completed the BREAST-Q questionnaire to assess subjective outcomes regarding psychosocial, physical, and sexual well-being, as well as breast-related comfort. Satisfaction with breast appearance, psychosocial well-being, and sexual well-being was comparable between the two groups, while the non-mesh group reported significantly higher physical comfort (**Table 5**).

**Table 5:** BREAST−Q scale scores of patients.

Variable	PBR without mesh (N = 128)	PBR with mesh (N = 118)	P Variable
Physical well−being: chest	31.00(27.00,32.00)	27.00(25.00,33.25)	0.005
Psychological well−being	39.50(38.00,41.00)	39.00(37.00,40.00)	0.092
Satisfaction with breasts	54.00(53.00,56.00)	53.50(52.00,55.00)	0.051
Sexual well−being	19.00(18.00,20.00)	30.00(28.00,36.00)	0.179

BREAST-Q^®^ version 2.0 ^©^ Memorial Sloan Kettering Cancer Center and The University of British Columbia, 2017. PBR Prepectoral Breast Reconstruction;.

### 3.5 Cost analysis

#### 3.5.1 Direct costs

##### 3.5.1.1 Mesh cost

In the mesh group, [TiLoop Bra soft tissue reinforcement mesh; pfm medical, Germany] was used. Taking the most commonly used and lowest-priced TB10 as an example, the cost was 17,900 CNY per piece (approximately 2,453 USD/piece; size: 78.5 cm²). This cost was omitted in the non-mesh group.

##### 3.5.1.2 Implant cost

Both groups used the same prosthesis (Mentor MemoryGel Xtra Breast Implants; Mentor Worldwide LLC, USA) at a cost of 14,940 CNY per implant (approximately 2,100 USD/implant), with no difference between groups.

##### 3.5.1.3 Other consumables

No significant differences were observed in the consumables required between the two surgical techniques.

#### 3.5.2 Indirect costs

3.5.2.1 Operative time

The operative time was reduced by 17.5 minutes in the non-mesh group compared with the mesh group (107.50 [95.00–120.00] min vs. 125.00 [115.00–135.00] min, p<0.001). Although the exact cost per minute of operating room use could not be calculated, the longer operative time in the mesh group was associated with significantly higher operating room expenses.

3.5.2.2 Length of hospital stay

As the study center is a high-volume hospital (more than 10,000 surgeries have been performed each year for the past three years), a pre-admission policy was implemented, with all preoperative examinations completed in the outpatient setting. Patients were generally admitted one to two days before surgery and discharged on postoperative day 3 in the absence of complications. Wound dressing changes and drain removal were performed in the outpatient clinic. Therefore, no significant difference in length of hospital stay was observed between the two groups.

#### 3.5.3 Comparison of total costs

The total direct medical cost was significantly lower in the non-mesh group compared with the mesh group (42,242.36 [39,999.14–44,628.14] CNY vs. 60,083.01 [57,592.06–63,443.36] CNY, p<0.001). The cost difference was primarily attributable to the expense of the mesh material (17,900 CNY).

#### 3.5.4 Sensitivity analysis

The primary cost difference between the two groups was the expense of the mesh. To evaluate the impact of mesh price fluctuations on cost-effectiveness, we assumed that the mesh cost varied within a reasonable range while other costs (operative time, hospitalization, etc.) remained unchanged. Since the non-mesh group did not incur this expense, it consistently maintained a cost advantage.

## 4 Discussion

In recent years, the incidence of breast cancer has shown an increasing trend and a younger age of onset. Traditional radical surgery often results in organ and functional loss as well as psychological trauma, which profoundly affect patients’ quality of life. With continuous advances in comprehensive breast cancer treatment and related research, the overall prognosis of breast cancer patients has significantly improved, accompanied by growing demands for postoperative body integrity and quality of life. Thus, how to ensure oncological safety while maximizing breast form and function restoration through precise reconstruction techniques, thereby alleviating psychological burden, has become a major focus in breast surgery. In this study, 246 patients undergoing prepectoral implant-based breast reconstruction after mastectomy were included. Postoperative complication rates and aesthetic outcomes (except for chest wall health) were comparable between the mesh and non-mesh groups. Notably, prepectoral implant-based reconstruction without mesh reduced both patient and healthcare costs, and proved to be a safe and feasible option in selected patient populations, representing an ideal reconstructive approach.

In 1971, Snyderman and Guthrie first reported the immediate subcutaneous placement of silicone breast prostheses following mastectomy (5). However, this approach was gradually replaced by the subpectoral approach in the late 1970s due to high rates of prosthesis loss and displacement, severe capsular contracture, and unsatisfactory cosmetic outcomes (13, 17). The 1980s and 1990s represented the golden era of subpectoral breast reconstruction. Nevertheless, this technique was associated with common complications, including chest wall pain, motor dysfunction, and breast distortion caused by muscle contraction (17–19). In the early 2000s, the introduction of acellular dermal matrix (ADM) facilitated the resurgence of prepectoral reconstruction. Multiple studies have confirmed the safety of prepectoral breast reconstruction, most of which rely on mesh support (7–9, 11–13). Mesh-assisted prepectoral reconstruction simplifies surgical procedures, reduces animation deformity and capsular contracture, and accelerates postoperative recovery. However, it also has limitations, such as an increased risk of infection and skin necrosis, requirements for adequate subcutaneous fat thickness, and higher material costs. Furthermore, mesh-assisted subpectoral reconstruction fails to improve patient satisfaction. These drawbacks have prompted researchers to explore mesh-free strategies (13–16, 20, 21). Nevertheless, mesh-free prepectoral reconstruction still faces unresolved challenges, including limited evidence from high-quality studies, lack of consensus on minimum subcutaneous fat thickness, scarce long-term data in patients receiving postoperative radiotherapy, and higher risks of implant visibility, rippling, wound dehiscence, and implant exposure due to reduced tissue coverage. These issues necessitate careful preoperative evaluation and meticulous surgical technique. Emerging approaches, such as endoscopic- or robot-assisted mastectomy with immediate implant reconstruction, may help mitigate these risks. Future directions also include technical refinements, such as combining fat grafting to improve soft tissue coverage, along with long-term outcome studies.

One of the key goals of implant-based breast reconstruction is to improve patients’ quality of life. Tools such as the BREAST-Q questionnaire (including satisfaction with breasts, satisfaction with outcome, psychosocial well-being, sexual well-being, and physical well-being) allow direct comparison of how different reconstruction approaches impact patient satisfaction and daily life. In this study, no significant differences were observed between the two groups in terms of satisfaction with breasts, psychosocial well-being, or sexual well-being. However, the non-mesh group scored higher in physical well-being related to the chest. These findings suggest that mesh-free prepectoral implant-based breast reconstruction may help patients achieve better quality of life and facilitate recovery of social functioning.

### 4.1 Postoperative complications and prognosis analysis

In the non-mesh group, 4 patients developed wound infections (3.13%), compared with 6 patients (5.08%) in the mesh group, indicating a lower infection rate in the non-mesh group. Most cases improved with antibiotic therapy and wound care. However, 3 patients in the non-mesh group and 4 patients in the mesh group ultimately experienced wound dehiscence, necessitating implant removal.

At the end of follow-up, the incidence of severe capsular contracture was 2.34% in the non-mesh group and 4.24% in the mesh group. Although the mesh group had a higher rate of capsular contracture, the difference was not statistically significant. This finding differs from previous studies (7, 8, 22, 23) and may be related to several factors: ①Foreign body reaction: The mesh acts as an additional implant, potentially eliciting a stronger foreign body response, promoting fibrosis, and increasing the risk of capsular contracture. Some studies have reported that mesh resorption and remodeling may be accompanied by chronic inflammation. In the non-mesh group, only the implant is present, eliminating one potential source of inflammatory stimulation and possibly reducing fibrosis (24). ②Mechanical distribution: In the non-mesh group, the implant is in direct contact with subcutaneous fat and fascia, which may facilitate the formation of a more uniform capsule and reduce contracture caused by uneven mechanical stress. In contrast, uneven mesh fixation may alter local mechanical distribution, leading to uneven stress on the implant surface and promoting contracture. Some literature also suggests that when the mesh does not fully cover the implant, uncovered areas are at higher risk for contracture (8, 16). ③Infection and seroma risk: Mesh may increase the risk of infection and seroma formation due to larger surgical trauma and retained foreign material. Infection is a well-established trigger for capsular contracture. In the non-mesh group, the simpler procedure reduces the likelihood of seroma and chronic inflammation, indirectly lowering the risk of contracture (23). However, the follow-up period in this study was relatively short, and confounding factors such as smoking and diabetes were not fully excluded, which may contribute to the difference from previous findings.

Complications commonly reported in prepectoral breast reconstruction, such as implant rotation, rippling, and implant visibility, were not evaluated in this study. The reasons are as follows: no cases of implant rotation were reported during the follow-up period; furthermore, the incidence of patient-reported rippling and implant visibility was extremely low. In addition, the moderate/severe rippling reported by most patients was inconsistent with the clinical observations. In future studies, we will prolong the follow-up duration and add standardized physician assessment of these indicators during outpatient visits to obtain further research outcomes.

Further subgroup analysis of patients who received postoperative radiotherapy showed a trend toward a higher incidence of severe capsular contracture in the mesh group compared with the non-mesh group (20% vs. 7.7%, p=0.193). This may be related to the following factors: ①Synergistic amplification of dual fibrotic stimuli: Radiotherapy activates pro-fibrotic pathways, such as TGF-β signaling, driving myofibroblast activation and collagen synthesis. The mesh, as an implant, elicits an inherent foreign body reaction and chronic inflammation during integration. These two strong pro-fibrotic stimuli may act synergistically rather than additively, exacerbating excessive fibrous capsule formation and contracture around the implant. ②Radiotherapy-induced tissue ischemia and fibrosis: Radiation can impair tissue perfusion and promote fibrosis, potentially accelerating or aggravating mesh contraction, which applies abnormal or uneven mechanical stress to the underlying implant. This persistent mechanical stimulus is a key driver of capsular contracture (25–29). In the non-mesh group, although the incidence of severe contracture was lower, it remained relatively high. While the mesh-free technique avoids additional foreign body reactions and mechanical risks associated with the mesh, radiotherapy still directly damages subcutaneous soft tissue and the microvascular network, resulting in tissue hypoxia, local ischemia, and a chronic inflammatory state conducive to contracture formation (29, 30). The difference between the two groups in this study was not statistically significant, likely due to the small sample size and relatively short follow-up. Nevertheless, the observed incidence of severe capsular contracture in mesh-reconstructed patients receiving radiotherapy warrants careful attention. For patients requiring postoperative radiotherapy, a staged approach—using a tissue expander initially and replacing it with an implant after radiotherapy—may be a more feasible strategy.

Long-term follow-up studies over 5 years have shown that patient-reported satisfaction in the non-mesh group was more stable than in the mesh group (mean score decline: 3.2 ± 1.8 vs. 7.5 ± 2.3 points, p=0.02), with the difference being even more pronounced in the radiotherapy subgroup (p=0.005) (30). Therefore, for patients scheduled to receive postoperative radiotherapy, the “mesh + implant” approach should be carefully considered. At the study center, a staged strategy—initial implantation of a tissue expander followed by replacement with a permanent implant after radiotherapy—is more commonly adopted. For patients requiring immediate implantation, a mesh-free approach may be the more suitable option.

### 4.2 Perioperative characteristics analysis

This study found a significant difference in operative time between the two groups, with the non-mesh group having a shorter operative time (107.50 [95.00–120.00] min vs. 125.00 [115.00–135.00] min, p<0.001) (**Table 2**). This finding is consistent with multiple international studies, and the additional time in the mesh group is mainly attributable to mesh trimming and fixation (19). There was no statistically significant difference in total drainage volume during the first three postoperative days between the two groups (202.99 ± 60.55 vs 198.44 ± 54.48, p=0.620). Intraoperative blood loss was slightly higher in the mesh group (50.00 [50.00–70.00] mL vs. 60.00 [45.00–75.00] mL, p=0.081), but the difference was not statistically significant (**Table 2**). This observation challenges earlier assumptions that mesh placement significantly increases bleeding risk (15) and may be related to improved understanding of peribreast ligament anatomy, preservation of the fascia, and increased surgical proficiency.

### 4.3 Satisfaction analysis

Patient-reported outcomes, including breast satisfaction, psychosocial well-being, chest physical status, and sexual well-being, were assessed using the BREAST-Q questionnaire. In the domain of Physical well-being: chest, the non-mesh group scored significantly higher than the mesh group (31 (28, 30) vs. 27 [25–33.25], p=0.005), consistent with multiple international studies, as patients in the mesh group more frequently reported sensations of “foreign body” and “restricted muscle movement” (17). Scores for Satisfaction with breasts, Psychological well-being, and Sexual well-being were comparable between the two groups (**Table 5**).

Under the premise of ensuring oncological safety, prepectoral implant-based breast reconstruction achieves excellent aesthetic outcomes. The non-mesh group demonstrated higher Physical well-being: chest scores and a trend toward lower capsular contracture rates, which may further contribute to improved patient quality of life.

### 4.4 Cost analysis

This study partially quantified the cost advantage of mesh-free prepectoral breast reconstruction. The results showed that total hospitalization costs were significantly lower in the non-mesh group compared with the mesh group (42,242.36 [39,999.14–44,628.14] CNY vs. 60,083.01 [57,592.06–63,443.36] CNY, p<0.001) (**Table 2**), while patient-reported outcomes for breast satisfaction, psychosocial well-being, and sexual well-being showed no significant differences. Notably, the cost of the mesh accounts for approximately one-quarter of the total expense in conventional prepectoral reconstruction. Mesh-free implant-based breast reconstruction thus offers a more feasible option in regions with limited medical resources, such as low- and middle-income countries.

Although this study did not perform a formal sensitivity analysis, the non-mesh group avoided mesh-related costs, and the mesh accounts for a substantial proportion of total expenses. Even if mesh prices were to decrease, the mesh-free approach would still reduce overall medical costs, indicating that its cost advantage is robust against market fluctuations. This finding aligns with the “selective patient model” proposed in previous studies: for patients with subcutaneous fat ≥1 cm, omitting the mesh does not increase complication risk while significantly reducing the burden on the healthcare system. In the context of cost-containment policies, mesh-free techniques may represent a more cost-effective option (31–33). Some studies have suggested that although mesh-free implant-based breast reconstruction reduces the direct costs of the primary surgery by avoiding expensive biological materials, it may be associated with a relatively higher rate of unsatisfactory aesthetic outcomes and symmetry, thereby increasing the need for secondary revision procedures. The costs related to these revision surgeries, such as fat grafting, implant replacement, or even conversion to mesh-assisted reconstruction, may partially or completely offset its initial cost advantage. Therefore, a long-term perspective is required when comprehensively evaluating the economic efficiency of this technique, and the costs and probabilities of subsequent interventions should be incorporated into the cost–benefit analysis model (34–36). In addition, this cost–benefit balance may vary substantially across different healthcare systems. Future research should collect multicenter cost data, extend follow-up periods, and establish cost prediction models adapted to different payment systems, so as to verify the generalizability of the cost advantages of the mesh-free strategy.

### 4.5 Study limitations

This study has several limitations. First, no subgroup analysis was performed for different types of meshes. Second, implant rotation, rippling, and implant visibility were not assessed, and no comparison was made regarding drainage duration and total drainage volume. In addition, the cost analysis was based on a single institution and may not be applicable to other healthcare systems. The follow-up period was relatively short, and the cost impacts of different mesh materials were not compared. Furthermore, the sample size of the radiotherapy subgroup was small. All these issues need to be further investigated in future studies.

## 5 Conclusion

In conclusion, this study demonstrates that compared with prepectoral prosthetic breast reconstruction with mesh, prepectoral prosthetic breast reconstruction without mesh reduces initial surgical costs and is clinically feasible in appropriately selected patients without significant differences in the incidence of major complications. Rational patient selection and long-term follow-up evaluation will help to further clarify long-term economic and clinical benefits. Notably, in patients undergoing postoperative radiotherapy, a high-risk population for Baker III/IV capsular contracture, the non-mesh group exhibited a lower rate of capsular contracture. This finding provides an important basis for surgical selection in radiotherapy patients: immediate placement of a tissue expander followed by exchange for a permanent prosthesis after completion of radiotherapy may be a more feasible strategy. Patient-reported satisfaction further validates the advantages of the non-mesh technique in terms of physical comfort. This study confirms that non-mesh prepectoral prosthetic breast reconstruction is a safe, feasible and cost-effective surgical option in a specific patient population. It shows positive significance in reducing medical expenses and simplifying surgical procedures, and has the potential for popularization and application, especially in clinical settings emphasizing medical cost control. Further large-sample and long-term follow-up studies are warranted to improve the evidence base.

## Data Availability

The original contributions presented in the study are included in the article/supplementary material. Further inquiries can be directed to the corresponding authors.
